# Some Recent Cases of Appendicitis*Read at September Meeting, 1899, Austin District Medical Society.

**Published:** 1899-10

**Authors:** T. J. Bennett

**Affiliations:** Austin, Texas


### TEXAS MEDICAL JOURNAL.

ESTABLISHED JULY, 1885.

PUBLISHED MONTHLY.—SUBSCRIPTION $1.00 A YEAR.

VOL. XV. AUSTIN, OCTOBER, 1899. No. 4.

### Original Contributions.

For the Texas Medical Journal.

### Some Recent Cases of Appendicitis.*

BY T. J. BENNETT, M. D., AUSTIN, TEXAS.

The New York Post-Graduate School showed the class last winter about seven or eight appendectomies a week, about twenty-five per cent. of which were pus cases, the balance primary or chronic. Public sentiment up there demands an early operation, when it is simple and comparatively free from danger. In this part of the country the operation is either deferred by the physician in charge or objected to by the patient or his friends, and the result is the majority of cases which are operated upon here are of the graver class, are pus cases, with more or less extensive adhesions complicating. The physician defers the operation because he believes the patient will recover without it, and that there will be less risk than with his operation. We cannot, all of us, become experts, because it is impossible to obtain the necessary experience. It is also impossible to obtain the services of an expert in all parts of the country. We must do the best we can for the patient, and if our operation would not give the patient a better chance than nature with our assistance could give, we had better trust to the "sublimity of luck." The radical advice, therefore, given out by such men as Morris and Deaver, to operate on all

*Read at September Meeting, 1899, Austin District Medical Society.

cases as soon as the diagnosis is made, does not seem to me to be just the thing under all circumstances, unless, possibly, such men could be at hand to do the work. Cases without complications are simple enough. Any clean operator should have no hesitancy in promising good results. But the cases with extensive peritonitis and adhesions and with perforation and pus are liable to run up his mortality record. It is impossible to describe the operation in detail. I mean that part of it which refers to the breaking down of adhesions.

The surgeon introduces his fingers into the abdominal opening and feels very little that he recognizes. The inflammation, adhesions and oftentimes misplacement of the viscera, make the search a blind one, and if the opening be so extended that he can see the parts, the situation is not often improved. The educated finger knows how to meet the difficulty. The adhesions are broken down and you know how to do it; the finger may move along here, and be gently insinuated there, and in time the delicate structures are liberated without damage and the "bird" is lifted into view.

In practically all such cases perforation and leakage have taken place, and if the leakage is small and the peritoneum can control the perforation and can take care of the debris, the case will most probably recover in five or six weeks. But if these conditions are exaggerated, the hole in the appendix is larger, or is reinforced by another perforation, this time in the bowel, and the contents poured out is greater than can be controlled, the case is practically hopeless without surgical interference. I need not mention the different methods of disposing of the stump of the appendix. It suffices when the freshened parts are stitched together and covered with peritoneum. It is a mistake to tie the mucous surfaces together, for they will not unite. The peritoneum, however, may come to our relief and finish the job for us by covering over the stump in its own peculiar way. We may congratulate ourselves then that we have made a success, that we have done well, but the truth is, we only had a better assistant than we reckoned. The peritoneum is certainly great. It is the silent assistant who never boasts of the part he plays, and saves our "mutton" in many a pinch. Give the peritoneum anything like a fair showing and it will "abate the nuisance;" it will take care of any ordinary amount of pus, such as may be left in the abdominal cavity after wiping out with gauze or irrigating, provided the source is controlled and the infection has not extended over more than one-fourth or one-half the peritoneal surface. Morris says the peritoneum can digest a steak. A local leucocytosis is the thing aimed at by the peritoneum. A volunteer army of leucocytes is thus lined up against the foreign enemy, the colon bacillus and pus organisms, and a battle to the death is waged for supremacy. Peritonitis, then, is a conservative process.

The six recent cases, which I thought might interest the society, consist of four operative and two treated tentatively. Of the four operated on two were pus cases and two primary. The first was a lad, sixteen, who gave no history of previous attacks. He was taken in June with severe crampy pains in the middle abdominal region. Dr. Mathews, who had the case in charge, described the condition as painful, requiring several doses of morphia each day to keep the patient at all comfortable. The bowels had been thoroughly emptied by salines or oil, and at the time of my first visit the right iliac region showed rigidity of muscles and a slight tumefaction. The temperature was not high, 102° I believe, and the pulse, while not so rapid as at Dr. Mathews' morning visit, it was considerably quickened. A liquid diet, including peptonoids and sufficient of the opiate to control pain, were continued as had been previously directed. At the visit next morning all of the previous severe symptoms had become ameliorated and the patient, with a nearly normal temperature, was considered so much improved that the question of an operation was indefinitely postponed. The patient continued to grow better until he was able to be out on the street. The spasm of muscle and tenderness in the right side, however, did not entirely disappear. This condition continued for two months, July and August. In the meantime Dr. Mathews had gone out of town, and the case had fallen into my hands. About the first of September, the patient became worse, the temperature rose, profuse sweats and increased tumefaction and pain made it necessary to undertake an operation.

An opening over McBurney's point brought into consideration several ounces of a very offensive yellowish fluid and a mass of adhesions too conglomerate for description. Pads of gauze were placed in the abdominal cavity to protect the uncontaminated peritoneum. The adhesions were broken down with great difficulty, and the appendix brought out. It was in a sloughing condition, enlarged and perforated. I removed it after no special rule on account of the vile condition it was in, and by much stitching I secured a fair closure of the stump opening. There was also a perforation in the bowel, about two inches back from the appendix. This opening was circular, with ragged edges, and was as large or larger than a two-bit piece. I simply darned up this hole as a woman would a hole in a stocking. The inflamed, weakened tissues would not allow any other method of closure.

After mopping and irrigating the locality, I placed this portion of the colon near the external opening so as to favor free exit of contents should union fail at the part stitched. An inch and a half of external opening was left for drainage with tube and gauze. That night the patient vomited a great deal, and in the early morning the nurse discovered that quite a quantity of bowel contents were passing from the wound. There was increased swelling and so much pain that I thought best to reopen and investigate. This revealed a large round opening in the colon five or six inches above the end of the bowel. It was from this opening that the bowel contents were issuing, and not from the hole stitched up on the day before as I feared it might be. I darned this opening in the same manner as I had done the other, and left the stitched part in the same relation with reference to drainage as I had left the other. But the stitches did not hold, and in three or four days fecal matter was passing from the wound as before, but in less quantity. Adhesions, however, took place promptly and in a manner to force all the fecal matter out at the wound, and so I left things until nature in her own inscrutable way brought about closure of the bowel opening, and complete restoration of the patient, some five or six weeks later.

The second case, J. H., was that of a young man, strong and healthy, 22 years of age. He gave no history of having had previous attacks. The onset was rather sudden, and very severe. The pain was great and required a grain and one-half to two grains of morphia in the twenty-four hours for relief. The temperature remained 102 and above. The bowels were kept open by salines. After forty-eight hours the symptoms had not improved, and it was decided to operate without further delay. There was nothing unusual about this case, except possibly in the operation itself; it was an early one, and at a time which could not have been further postponed without complications of adhesions and pus. A prompt and complete recovery followed the operation.

The third case was a patient of Dr. G. L. Robertson, of Leander. It was in many respects like the first case, except that it gave a history of several previous attacks,—a "recurrent" case.

A young man, about 20 years of age, was attacked suddenly with severe pains in the right iliac region. Tumefaction was soon recognized in the region of the appendix. The temperature ran from 101° to 104°. The patient suffered great pain. At the time of my visit the patient had been confined to his bed about two weeks. There were profuse sweats, variable temperature, and quite a tumor pointing in the iliac region, and bulging and great tenderness in the right lumbar region. Cutting down over the most prominent portion of the tumor, which was about over the McBurney point, a large quantity of pus and debris escaped—about sixteen ounces. The adhesions, as in the first case, were extensive and difficult to break down so as to lift out the appendix. Leakage, which had been suspected, was found to come from a perforation in the appendix near the middle. The appendix was excised after the method of Joe Price, "as you would cut the rot out of an apple." Owing to the sloughy condition of the tissues it was a question whether the stitches would secure union of the parts, and, in taking leave of the head of the colon, the stitched portion was placed near the external wound, as in the first case, to favor an easy escape of bowel contents in the event the stitches gave way. Gauze for drainage was placed in the wound. Within a few days, as was expected, the ligatures gave way and a fecal fistula was established. A knot of suture material (formalin catgut) passed out. The opening in the bowel closed in five or six weeks and the patient made a good recovery.

The fourth case was Dr. P., a rather slender young man of about thirty. He gave a history of two previous attacks, one about thirteen months and the second four months before. The first attack was very severe and lasted several weeks. The second was not so severe, but equally as painful for the length of time it lasted. Two or three days before I saw him a distressed feeling with occasional severe pains set up in the old region, and the doctor, wise man that he was, decided to part company with his appendix while times were good. The operation was uneventful. The stump of the appendix was ligated and covered over with muscularis and peritoneum. In ten or fifteen days the patient was out attending to business. The lumen of the appendix in this case was closed for about half its length, the distal end. The other portion contained a small amount of mucopus, but was not specially enlarged and apparently but slightly inflamed. No adhesions existed.

The fifth case was Capt. W., a corpulent man of fifty or more years. This was his first experience with appendicitis. The onset was rather sudden and very severe. The bowels were moved within the first thirty-six hours, and kept open with phosphate of soda. Rigid muscles in the right iliac region and a tumor larger than a hen egg were constant objective symptoms throughout the attack, which lasted, in the more acute stage, five or six weeks. A liquid diet for the most part was given. Very little medicine of any kind was employed. A teaspoonful of phosphate of soda, to which a little bicarbonate of soda had been added, was administered *pro re nata.* The temperature ranged between 100° and 102½°. At times there was profuse perspiration with a tendency to chilliness. Pus was no doubt present, but the leakage was evidently small and the peritoneum controlled it. This case might have been operated upon. It looked like it demanded the operation, but the patient was willing to divide the chances and trust to luck. He made what seems to be a good recovery. The question was whether further leakage would take place. It did not, but nobody knew this until time enough elapsed to show it.

The sixth case was very similar to the fifth. G. L. H., a young man, with a previous history good, was attacked with severe crampy pains in the hypogastric region. Vomiting, fever, rigid muscles in the right iliac region, and great pain marked the onset of what proved a very serious and prolonged attack of appendicitis. A small tumor could be palpated in a few days. Under the use of saline laxatives, opiates and various applications, ice bags, poultices, etc., the patient, at the end of the first week or ten days, was much improved. But a second leak evidently made its appearance, and renewed and aggravated symptoms followed. The tumor became larger, and for the next five or six weeks the patient suffered a great deal and was in great danger. This patient should have had the operation, but the first severe symptoms having materially subsided the risk of voluntary recovery was assumed. The patient did recover, but has lived since under great anxiety for fear of a second attack. He has had pains upon extra exertion on several occasions since the attack, due, I thought, to old adhesions. This man is ready for the operation upon the first real suggestion of a second attack.

The pus cases, above reported, are interesting because they are the kind we most frequently operate upon in this country, and because the greatest care and skill are required to give them a good prospect. I believe the chromacised or formalin catgut is the ideal buried suture. Another point I would emphasize in pus cases, especially where there are perforations to be stitched in bad tissue: and that is, to leave the stitched part as near the wound outlet as possible, not to displace the gut too much. This facilitates the exit of the bowel contents should the stitches not hold.

Seeds and other foreign substances are not any longer regarded as necessary in the causation of appendicitis. In fact, such substances are causative only when they contain disease producing bacteria, or when a traumatism has rendered the field a better culture medium for the growth of such germs. The true and direct cause of appendicitis, as accepted now by leading pathologists, is infection, produced by the colon bacillus and other germs and toxins which abound in greater or less quantities at all times in the alimentary canal.

The appendix is peculiarly constructed for harm. It is made up largely of lymphoid tissue, and therefore absorbs poisons readily; by its meagre muscular supply and its dependent position it can hardly get rid of anything that gets into it; guarded at its mouth by the valve of Gerlach, a mere little fold of peritoneum, which is a valve only in name, there is practically no resistance to the entrance therein of any toxic agent or germs that may be passing that way in the bowel. As a matter of fact the appendix is a functionless relic of an organ once useful in our now "renowned ancestors," and seems to be the only perfect cul'ure tube for the growth of pathogenic bacteria in our entire anatomy. Its only purpose, as waggishly expressed by some one, is "to have appendicitis"; and granting the foregoing, it is not hard to understand why appendicitis is so common.

We can readily see from the structure of the organ and its anatomical surroundings what devastation an infectious inflammation could work. The slight crampy pains noticed in the earlier stage of the disease are thought to be due to contractions of the muscular fibres of the appendix. The heavier pains, attended with perturbations of temperature and circulation, manifesting themselves suddenly, or even coming on slowly, are thought to be due to perforations and leakage in a greater or less degree. The peritoneum with its superhuman foresight and tact, endeavors to surround this foreign substance and disinfect it, digest it, and absorb it; otherwise, as Morris puts it, "nature will have to have a compliment paid her," and the organ removed. Much judgment, with careful watching, is necessary to determine the progress of a case of appendicitis. The pathology is now much clearer than it was only a few years ago. The careful practitioner can nearly always say that the case is growing worse or is at a standstill; that the fight the peritoneum is waging with the enemy is losing or gaining, and the spreading of the infection determines the question of an operation. This can be decided, in most cases, within the first thirty-six to forty-eight hours. At this time the adhesions are not formidable, and the pus or infected serum is not great.

The technic of experts is not always the same; but the results do not differ widely. It should be interesting here to contrast the methods of two great leaders in appendectomy; that of Dr. Robert T. Morris, of the New York Post-Graduate Medical School, and Dr. Jno. B. Deaver, of the University of Pennsylvania.

Morris makes a very small incision with a pair of scissors, extending through the skin and subjacent tissue to the muscles. Then with the points of the scissors he spreads the muscles apart and stretches the opening as wide as the slit in the skin. He uses his fingers to spread the opening after getting it started with the scissors. When the peritoneum is reached a thread is attached to it and left there to be used as a "guy-line" while further operating. A small incision, one-half or three-quarters of an inch long, is made in the peritoneum, and, as in the case with the muscles, this opening is stretched wide enough to permit the entrance of a finger or two into the abdominal cavity. The appendix is found, the adhesions broken down everywhere, and is lifted out. Absorbent gauze is employed to clean out the abdominal cavity, and where there is much pus, peroxide of hydrogen is used on the gauze. The appendix and parts of the bowel that are out are washed with sterilized water and then the appendix is treated by making a circular cut with the scissors within one-half inch of the bowel. This cut extends through the peritoneal and muscular layers and down to the mucous coat of the appendix. A circular stitch is then placed in position and the appendix tied off with another ligature and cut away below the top of the "puckering string." Then this circular or puckering string is drawn and tied, and a stitch or two more draws the peritoneum snugly over the stump, when it is dropped back into the abdominal cavity. Where the adhesions have been formidable, aristol is used to prevent their reformation. The next step is to draw on the guy-line, which brings into view the peritoneal coat to be stitched separately, with catgut. Then the muscles are stitched in the same way, and last a subcuticular suture closes the skin. A little aristol is now sprinkled on, and a fold of guaze is placed on and held by the two ends of the subcuticular suture being tied over it. Another piece of folded gauze is laid over this piece, and over this, a piece of cotton, which is held in position by adhesive strips. The operation is now complete; and the patient is put to bed for ten days, when (in some ninety-eight out of a hundred cases) he is permitted to "pick up his bed and walk."

Deaver, on the other hand, makes an incision three or four times as long as Morris does. He cuts all the way, and stretches nothing, not even his imagination; for the "bird," as he called the appendix on one occasion, is brought into plain view. He then packs the abdominal cavity with gauze so as to prevent contamination in the further steps of the operation. Ten to fifteen yards of gauze are not unusual for Deaver to use in a single case. You wonder where he could pack that amount of gauze, but he stuffs it in and around somewhere. Then he breaks the adhesions down, letting out the pus, if any be present, and treats the "bird" *a la* Joe Price. This methd does not differ so much from that of Morris. Deaver cuts the appendix out, including a little of the funnel-shaped cœcum, and stitches the peritoneum and muscular coats over the site. Deaver uses silk throughout, except he closes the external opening with silkworm gut. Morris uses chromacised or formalin catgut for everything.

